# *Helicobacter pylori* and Systemic Disease

**DOI:** 10.1155/2014/358494

**Published:** 2014-03-19

**Authors:** Chao-Hung Kuo, Yen-Hsu Chen, Khean-Lee Goh, Lin-Li Chang

**Affiliations:** ^1^Division of Gastroenterology, Department of Internal Medicine, Kaohsiung Medical University Hospital, Kaohsiung 807, Taiwan; ^2^Department of Medicine, Faculty of Medicine, College of Medicine, Kaohsiung Medical University, Kaohsiung 807, Taiwan; ^3^Division of Infectious Diseases, Department of Internal Medicine, Kaohsiung Medical University Hospital, Kaohsiung 807, Taiwan; ^4^Department of Medicine, University of Malaysia, 50603 Kuala Lumpur, Malaysia; ^5^Department of Microbiology, Kaohsiung Medical University, Kaohsiung 807, Taiwan

Currently,* Helicobacter pylori (H. pylori) *infection is confirmed to correlate with chronic gastritis, peptic ulcer disease, Mucosa Associated Lymphoid Tissue (MALT)-lymphoma, precancerous changes in the stomach (atrophy, intestinal metaplasia), and gastric cancer. At the same time,* H. pylori* eludes the immunological response evoked by the host. This chronic infection has the local production and systemic diffusion of proinflammatory cytokines, which may influence the remote organic systems and result in extragastric manifestations [1] (Table 1).

Several studies performed during the past years have supported the possible role for* H. pylori *infection in the pathogenesis of several extragastric diseases. The role of* H. pylori* in some hematologic conditions was included in the current guidelines, such as immune thrombocytopenic purpura (ITP), iron deficiency anemia (IDA), and vitamin B12 deficiency [2–4]. The effects on other systems such as cardiovascular diseases, diabetes mellitus, dermatological disease, and neurologic disorders have also attracted researchers' concern. Data known from those studies have shown that the immunological response caused by* H. pylori* might influence the clinical outcome of these diseases. However, many of these reports suffer from being case reports or case series without adequate controls.

The* H. pylori* eradication resulting in increasing the platelet count in adult patients with primary immune thrombocytopenia (ITP) has been confirmed [2, 4]. Moreover, there is sufficient evidence to regard* H. pylori* infection as a cause of unexplained sideropenic anemia (refractory IDA) by several mechanisms [3]. So, recent guidelines indicate* H. pylori* infection to be sought in IDA patients. Other hematological diseases possibly related with* H. pylori* included monoclonal gammopathy, megaloblastic anemia, and myelodysplastic syndrome [5].

Many previous studies stated that chronic infection with* H. pylori* has significant interactions with the immune system. Recent epidemiological data suggest that aggressively eradicating* H. pylori* infection might be related to an increase in autoimmune diseases [6], but the possible mechanisms remain controversial. Many researchers thought that* H. pylori* have acquired several abilities that help them escape clearance through the host immune system. Then* H. pylori* interacts with the immune system and results in its downregulation. However, controversial results were reported in several studies. We need further research studies focusing on the possible impact of* H. pylori* on autoimmune diseases.

The relationship between seropositivity for* H. pylori* and outcome of cardiovascular disease is also an important issue. Previous studies have surveyed the association between* H. pylori* infection and coronary artery disease (CAD) [7]. The possible mechanisms of* H. pylori* infection in the pathogenesis of CAD include persistent local or systemic inflammation and initiating autoimmune responses [8]. However, the level of supporting evidence is too limited to advocate therapeutic interventions. Accordingly, further randomized trials are needed to evaluate the role of* H. pylori* eradication in these patients.

Some studies have disclosed that the association of lung cancer risk with* H. pylori* infection is five to ten times stronger than with passive smoking exposure [9]. It raises the notion that* H. pylori* might be a risk factor among non-smoking-related lung cancer. Many possible hypotheses have been proposed including the following: (a) the mechanisms may vary by both* H. pylori* strain and subtype of lung cancer; (b)* H. pylori* infection status/eradication should influence the clinical outcome of lung cancer; and (c) this association should be influenced by other factors [10]. However, the possible mechanisms and evidence need more studies to confirm any of these.

The role of* H. pylori* in dermatological diseases is still a controversial subject. The association between chronic urticaria (CU) and* H. pylori* has been found by some research groups [11]. The evidence comes from studies demonstrating that many patients with CU received clinical improvement after* H. pylori* eradication [12]. But recent trials, utilizing the Grading of Recommendations Assessment, Development, and Evaluation (GRADE) approach, showed different results where the benefit of* H. pylori* eradication in patients with CU was weak [13]. Other skin diseases also show controversial results and need further survey.

One recent meta-analysis stated that Type 2 diabetes and insulin use in diabetic patients are significantly associated with a higher incidence of* H. pylori* eradication [14]. Previous studies revealed that higher serological positivity of* H. pylori* were noted in patients with type 1 diabetes (T1DM) and autoimmune thyroiditis (AT). In their results,* H. pylori* infection could be considered as an environmental trigger for development of AT in T1DM. They suggested that young patients with T1DM should be screened for* H. pylori* infection [15].

When discussing medical economic policy, the relationship between* H. pylori* and systemic disease needs more attention. The population-based strategies for* H. pylori* eradication in people with low prevalence are unlikely to be cost-effective, but this management might be necessary in people with high risk of developing systemic disease. The challenge we face is to investigate whether, at what magnitude, and in which direction,* H. pylori *may be linked to systemic diseases and in which populations. We hope that this special issue will be helpful in the possible pathogenesis of* H. pylori* related extragastric manifestations.

*Chao-Hung Kuo* *Chao-Hung Kuo* *Yen-Hsu Chen* *Yen-Hsu Chen* *Khean-Lee Goh* *Khean-Lee Goh* *Lin-Li Chang* *Lin-Li Chang*

## Figures and Tables

**Table 1:** The extragastric manifestation of *H. pylori* infection.

Involved extragastric system	Extragastric manifestations
Cardiovascular system	Atherosclerotic heart disease, cerebral vascular disease
Neurological system	Parkinson's disease, migraine
Hematological system	Immune thrombocytopenic purpura, iron deficiency anemia, Vit B12 deficiency anemia
Immunological system	Raynaud's phenomenon, Sjogren's syndrome
Dermatological system	Chronic urticaria, angioedema, alopecia areata
Endocrine system	Diabetes, autoimmune thyroiditis
Ear, nose, eye, and throat	Hyperemesis gravidarum, anorexia of aging, glaucoma, oral ulcers
Others	Halitosis, urethritis
